# Combined extraction and spray-freeze-drying of *Cannabis sativa Flos* provides stable lyophilizate particles of cannabinoids

**DOI:** 10.1186/s42238-025-00381-w

**Published:** 2026-01-06

**Authors:** Jan Kožák, José Ignacio Vázquez-Olvera, Kai Berkenfeld, Annika Rautenberg, Alf Lamprecht

**Affiliations:** 1 Department of Pharmaceutics, Institute of Pharmacy, University of Bonn, Gerhard-Domagk-Strasse 3, 53121 Bonn, Germany; 2 Université Marie et Louis Pasteur, EFS, INSERM UMR1098 RIGHT, Besançon, F-25000 France

**Keywords:** Spray-freeze-drying, Cannabis, Storage stability, Tetrahydrocannabinol, Cannabidiol

## Abstract

The objective of this study was to explore non-aqueous spray-freeze-drying (SFD) as a formulation strategy to stabilize and improve the dissolution of *Cannabis sativa* full extracts. The medicinal use of Cannabis sativa has strongly intensified over the last decade. Here, *Cannabis flos* full extracts prepared by tert-butanolic solutions with 25% solid content were directly processed via spray-freeze-drying (SFD) with different excipients to increase the storage stability of the 4 main cannabinoids: tetrahydrocannabinol (THC), cannabidiol (CBD) and their corresponding acid precursors (THCA and CBDA). A variety of pharmaceutical excipients, namely polyvinyl pyrrolidone (PVP), HPMC-AS, PEG 6000, cetyl alcohol, or shellac were investigated for their sprayability and ability to form spray-freeze-dried particles (with a constant 25% cannabis resin content). SFD formulations varied in median diameter between 230 and 400 μm, depending on the respective excipient. The long term-stability tests revealed that, in the pure dried extract, the four cannabinoids were almost entirely degraded after 6 months at 40 °C, while PVP and HPMC-AS SFD-particles achieved a significant improvement in chemical stability of the tracked cannabinoids at 25 °C and 40 °C. Preliminary dissolution tests revealed that particles made from PVP, PEG 6000, and HPMC-AS enhanced the dissolution rate of THC and CBD, reaching higher concentrations than the raw extract before plateauing. It was demonstrated that non-aqueous spray-freeze-drying can be an advantageous process to formulate heat-sensitive, water-insoluble extracts into solid-state products with improved stability, offering a promising platform for future oromucosal and potentially nasal product development, pending further deposition and performance studies.

**Supplementary Information:**

The online version contains supplementary material available at 10.1186/s42238-025-00381-w.

## Introduction

The use of medicinal cannabis is increasing in importance in a variety of indications (Whiting et al. 2015; Pagano et al. 2022). Cannabinoids are highly permeable through mucosal membranes, however, the bioavailability of cannabinoids upon oral administration is poor and variable (Poyatos et al. 2020), due to a combination of their poor aqueous solubility, food effect (Jarho et al. 1998; Koch et al. 2020), and a pronounced first-pass effect (Samara et al. 1988).

The pulmonary administration of the cannabinoids via smoking or vaporizing of cannabis preparations is advantageous for its rapid onset of action and for bypassing the liver first-pass effect. However, is associated with multiple drawbacks, beginning with diminished effectiveness due to the exhalation of a dose fraction, which depends on inhalation depth and duration of breath-holding. Additionally, the inhaled smoke contains toxic (e.g. carcinogenic) substances and foreign particles causing adverse effects, from acute irritation (cough) to chronic damage of the respiratory tract (e.g. bronchitis, lung cancer) (Tashkin 2005; Ribeiro et al. 2016; Tashkin et al. 2019; Hancox et al. 2019) being unacceptable in modern pharmacotherapy. Vaporizing resolves some of such adverse effects of smoking, but still such a way of administration is often considered as unacceptable for many patients due to the social stigma associated with cannabis recreational use, as it is difficult to apply doses in a discreet fashion in comparison with the conventional drug dosage forms (Reid 2020; Troup et al. 2022), with the inherent risk of affecting adjacent people, patients and healthcare personnel.

In many of the cannabis indications, e.g. alleviation of acute pain, a rapid onset of action is desirable. While this can be achieved, besides the pulmonary route, with oral liquid formulations, these have short shelf-lives due to the poor chemical stability of the cannabinoids in liquid solutions. Therefore, solid-state formulations that enhance both solubilization and stability parameters, represent suitable approaches to address these challenges in cannabinoids therapy. Spray Freeze Drying (SFD) is a powder engineering technique that combines atomization and solidification with subsequent sublimation, enabling the transformation of liquid extracts into highly porous, free-flowing powders. Compared to conventional drying methods, SFD operates under no thermal stress, making it particularly suited for heat-sensitive and chemically labile compounds such as cannabinoids (Van Drooge et al. 2005). The resulting features of such SFD powders allows enhancement in the solubility of poorly water-soluble drugs (Lucas et al. 2022; Kožák et al. 2025), and the improvement in the long-term storage stability of labile compounds (Serim et al. 2021). Furthermore, the multiparticulate constitution allows administration via alternative routes that bypass the first-pass effect such as the nasal and oral mucosa (sublingual/buccal), and because of the good membrane permeability of the cannabinoids, these routes can offer a high bioavailability as well as a rapid onset of action.

While pharmaceutical formulations usually only contain the main active compounds (THC and/or CBD; e.g. Sativex^®^), the whole extracts from the cannabis plant material is often preferred for the additional therapeutic benefits of the synergistic effects of the full spectrum of cannabinoids, as well as the accompanying terpenes and flavonoids (the so-called “entourage effect“) (Ferber et al. 2019; Cogan 2020). In this study, the general feasibility of formulation of a cannabis full extract resin-containing SFD product was investigated, including an evaluation of stabilizing impact of suitable excipients.

For this, we have investigated here the application of tert-butanol, a low molecular-wight and low melting point (+ 25 °C) solvent, as a single processing solvent for both the initial extraction of cannabinoids from the dry plant material and the subsequent SFD powder production.

Therefore, the choice of excipients for embedding the cannabis resin in the SFD product was restricted in this study to demonstrate sufficient solubility in tert-butanol and acceptable final mechanical integrity of the particles (proper structure forming properties). We aimed to investigate a broad variety of excipients of different physicochemical properties to screen their effect on their ability to prevent degradation of the incorporated cannabinoids as well as enhancing their aqueous dissolution. On the other hand, cannabinoids such as THC, CBD, and their acidic precursors are highly prone to oxidative degradation, which limits product shelf-life and quality. While SFD protects these compounds from thermal stress, residual oxidation remains a major challenge. The incorporation of antioxidants offers a targeted strategy to further enhance stability.

## Materials and methods

### Materials

Medicinal cannabis flowers, variety PEDANIOS 8/8 with 8% THC and 8% CBD content were purchased from AURORA Cannabis Enterprises Inc. (Cremona, Canada). The HPLC standards of CBD, CBDA, THC, and THCA at concentration 1000 µg/ml in methanol were obtained from Sigma Aldrich. Polyvinyl pyrrolidone (Kollidon^®^ 25) was a free sample from BASF (Ludwigshafen, Germany), cetyl alcohol and polyethylene glycol 6000 were obtained from Caesar & Loretz (Hilden Germany), shellac (SSB 55 grade) from HARKE Pharma (Mülheim an der Ruhr, Germany), Aquasolve^™^ HPMC-AS LF (hydroxypropyl methylcellulose acetate succinate) from Ashland Specialty Ingredients (Wilmington, USA). Tert-butanol, ascorbic acid, ascorbyl palmitate, and butylated hydroxytoluene were purchased from Carl Roth (Karlsruhe, Germany).

#### Excipient selection

A variety of pharmaceutical excipients with a broad range of physicochemical properties were chosen, such as polyvinyl pyrrolidone (PVP), hydroxypropyl methylcellulose acetate succinate (HPMC-AS), and polyethylene glycol 6000 (PEG) as hydrophilic polymers. Shellac as a nontoxic, widely used excipient in pharmaceutics, mainly used due to its impermeability to water vapour and oxygen as a protective tablet film-coating for sensitive drugs. Cetyl alcohol (CA) was further chosen as a lipophilic excipient as it can possibly provide better incorporation and hence stabilisation of the alike lipophilic actives.

Additionally, three potential antioxidative candidate have been chosen to be tested in the formulations: Ascorbic acid acts as a hydrophilic radical scavenger, ascorbyl palmitate provides protection at aqueous–lipid interfaces due to its amphiphilic nature, and BHT (butylated hydroxytoluene) stabilizes lipophilic environments typical of resin-rich extracts (Niki 1991; Yehye et al. 2015; Imran et al. 2024). Their different modes of action make them promising excipients to investigate oxidative degradation in SFD cannabis formulations and extend storage stability.

### Methods

#### Tert-butanolic cannabinoid extraction

In the plant material, the active substances THC and CBD are predominantly present as inactive precursors in form of their corresponding carboxylic acids (i.e. THCA and CBDA). These acids need to be decarboxylated to form the pharmacologically active THC and CBD, mostly performed through exposition to heat (Reason et al. 2022). The *Cannabis flos* plant material was heated before the extraction procedure in a closed container at 120 °C for 30 min to induce the decarboxylation reaction forming THC and CBD form respectively. Temperature and duration were selected based on preliminary kinetic studies (data not shown) where we tested various time-temperature combinations (100–140 °C, 15–60 min) and monitored THCA/CBDA conversion by HPLC, in order to achieve approximately 50% decarboxylation and of the initial THCA and CBDA content to investigate also the decarboxylation of THCA and CBDA to THC CBD respectively in the final product during the long-term stability study.

Because of the poor aqueous solubility of the cannabinoids, organic solvents are used for their extraction from the *Cannabis flos* and for further processing. Low molecular weight alcohols such as ethanol or methanol are standardly used (e.g. in Pharmacopoeia Europaea). Further commonly used solvents include hexane and liquefied propane or butane, which are, however, flammable and explosive and need to be fully removed from the final product Lazarjani et al. 2021). But none of such organic solvents has the specific characteristics required for the pharmaceutical freeze-drying (either too low freezing point, corrosiveness, or high toxicity). The non-aqueous freeze-drying is limited to very few suitable organic solvents, where the most common is tert-butanol. Tert-butanol being a low molecular weight and high melting-point alcohol (as the standardly used methanol and ethanol), was chosen as a suitable extraction solvent.

The ratio of *Cannabis flos* to the extraction solvent (tert-butanol or ethanol as a reference extraction solvent) was 1 g per 10 ml. The plant material was crushed and extracted in 3 cycles of 15 min in a sonication bath with 1 h stirring agitation in between. The plant material was separated by centrifugation followed by filtration through a PTFE membrane filter of 0.45 μm pore size. The total solid content in the final tert-butanolic extract was determined gravimetrically: 1000 µl of the extract was transferred in glass vials and solvent left to evaporate (*n* = 3), the dried residual was weighed on an analytical balance. The amount of the cannabinoids of interest (THC, CBD, THCA, CBDA) in the extract was determined by liquid chromatography.

#### Spray-freeze-drying

The tert-butanolic extract from the *Cannabis flos* was further entrapped in SFD particles (cannabispheres). Carrier excipient, i.e. either PVP, HPMC-AS, PEG 6000, cetyl alcohol or shellac, was added to the tert-butanolic *Cannabis flos* extract to yield a final w/w ratio of 25:75 extract: excipient and an overall concentration of 10 g/100 ml, except the HPMC-AS formulation solution that required dilution to 5 g/100 ml because of its too high viscosity for spraying using this nozzle setup. The PEG 6000 is insoluble in pure tert-butanol, therefore water was added in order to yield 90:10 (v/v) tert-butanol: water mixture. This ratio was a compromise to provide sufficient solubility of the water-insoluble cannabis extract and the tert-butanol-insoluble PEG 6000.

The solutions were spray-frozen using a one fluid nozzle (droplet generator, FMP Technologies GmbH, Erlangen, Germany) via 170 kPa pneumatic pressure. The nozzle was equipped with a 200 μm diameter orifice nozzle through which the forming droplets entered an approx. 1 m long cylindrical tower, cooled to -100 °C by an outer liquid nitrogen jacket – a process developed in our department earlier (Eggerstedt et al. 2012). The generated droplets froze during free fall which leads to the formation of frozen microspheres. The frozen microspheres were collected at the bottom of the tower in a glass vessel which was subsequently transferred to an Alpha 1–4 LSC Plus freeze dryer (Martin Christ, Germany) and dried at 0.05 mbar and 20 °C for 24 h. Pure extract was freeze-dried (in brown-glass vials) in parallel using the same conditions as the SFD particles to produce a pure dried resin that was used as a control.

#### Particle size and morphology

The appearance of the microspheres was investigated using a scanning electron microscope Hitachi SU3500 (Tokyo, Japan) at 5 kV. The samples were sputter coated with a thin gold layer (Polaron SC7640 Sputter Coater, Quorum Technologies Ltd., Newhaven, UK). Images were taken in nominal magnifications of 50, 200 and 400x. The median particle size was analysed by dynamic image analysis (DIA) using a Camsizer X2 system (Retsch Technology GmbH, Haan, Germany).

#### Liquid chromatography

The cannabinoids were quantified using an HPLC Waters 2695 chromatography system with a Waters photodiode array 996 detector (Waters Alliance, Milford, USA). The separation conditions were adapted from (Peschel et al. 2015). The column used was LiChrospher-100, 250 × 4.6 mm, RP18, 5 μm EC (CS-Chromatographie, Merck). Gradient flow was used for elution with a flow rate of 0.9 ml/min. Solvent A: acetonitrile; Solvent B: water-acetonitrile (65:35, 0.1% trifluoroacetic acid) with the following gradient: solvent B: 0 min 70%, 30 min 35%, 43 min 5% 48 min 70%; total sample running time 65 min. The UV detection was performed at 219 nm. The HPLC method had a linearity range of 20 to 150 µg/ml and limits of quantification were below 1 µg/ml for THC, THCA, CBD, and CBDA respectively.

#### Gas chromatography

The residual tert-butanol content in cannabispheres upon the freeze-drying was determined using a gas chromatography technique (FOCUS GC; Thermo Finnigan, USA) via a method established earlier (Lucas et al. 2022). Briefly, the conditions were as follows: The headspace vials with 20 mg of particles dissolved in 1.0 ml of 20/80 methanol/water were incubated in the autosampler at 80 °C for 30 min before injection. The syringe temperature was 120 °C. Fused-silica capillary column FS-CS-624 30 m x 0.32 mm I.D., 1.8 μm film thickness (CS-Chromatographie Service, Germany) was used for the separation, with nitrogen as carrier gas at 2 ml/min flow rate; a split flow of 10 ml/min with a total split ratio of 5. The injector temperature was 200 °C, detector 230 °C; the oven temperature was held for 10 min at 50 °C and subsequently increased to 200 °C at a rate of 25 °C/min.

#### Long-term stability

To investigate the long-term stability, the products were stored in closed brown-glass vials at 4 °C, 25 °C, and 40 °C, protected from light and analyzed at 1, 3, 6, and 12 months. The change in content of the four cannabinoids (THC, CBD, THCA, CBDA) was quantified using the liquid chromatography method described above. 20 mg of cannabispheres/freeze-dried product was dissolved in 3.0 ml of methanol (*n* = 3). Results are expressed as relative content in comparison with the fresh sample (t_0_), where the content at t_0_ was set to 100%.

Since the majority of the studies have so far focused only on the stability of THC and CBD in pharmaceutical preparations, we further expanded the study to the stability of the corresponding carboxylic acids THCA and CBDA as their interplay with THC and CBD concentrations can elucidate degradation during storage in more detail.

#### In vitro dissolution

The dissolution of 10.0 mg of cannabispheres or 2.5 mg of crude resin was performed in 0.05 M phosphate buffer pH = 7.0 containing 0.1% polysorbate 80 (*n* = 3). The volume of the dissolution medium was 5.0 ml in order to better mimic the low liquid volumes upon sublingual/nasal administration (Ìkinci et al. 2004); the temperature was 37 °C and stirring speed 200 rpm (magnetic stirrer, 8 mm length). Samples (0.1 ml) were withdrawn at 5, 15, 30, and 60 min. The dissolved THC and CBD were quantified using liquid chromatography.

Crude cannabis extract was incubated in the dissolution medium for 48 h hours and 3.3 ± 0.4 µg/ml of THC; 2.9 ± 0.3 µg/ml of CBD were considered as saturation solubility under these test conditions.

#### Statistical analysis

The effect of excipient on both dissolution performance (percentage of THC and CBD dissolved at 60 min) and chemical stability (relative endpoint content of THC, CBD, THCA, and CBDA under each storage condition) was analyzed using independent one-way analyses of variance (ANOVAs). Assumptions of normality and homogeneity of variances were assessed using the Shapiro-Wilk and Levene’s tests, respectively. When significant differences were detected, post-hoc pairwise comparisons were performed using Tukey’s Honestly Significant Difference (HSD) test. Effect sizes were calculated using Cohen’s d to quantify the magnitude of differences between excipients. All statistical analyses were conducted in RStudio (version 2025.05.1 Build 513) using the packages car and agricolae.

## Results

### Extraction with tert-butanol

**Fig. 1:**
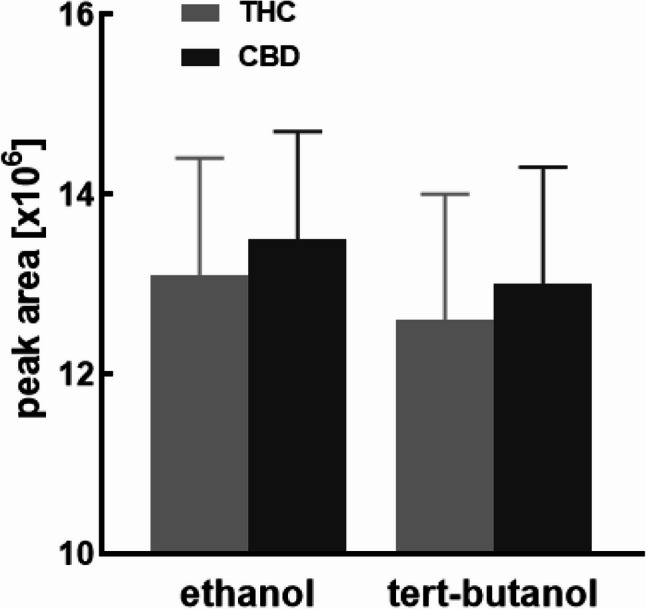
The extracted amount of THC and CBD using tert-butanol was around 97% of the amount using ethanol, however differences were not statistically significant (data are given as means ± SD; *n* = 3)

The solid content in the case of tert-butanolic as well as ethanolic extract was found to be approx. 3% (w/v). There was also no statistically significant difference in the extracted content of cannabinoids (THCA, THC, CBDA, and CBD) between the tert-butanol and ethanol extraction solvents (Fig. 1). The THC and CBD content in the dry extract resin prepared via tert-butanolic extraction and used in the SFD formulations was determined to be 15.5 ± 0.1% THC and 9.3 ± 0.1% CBD.

### SFD particle characteristics

**Fig. 2:**
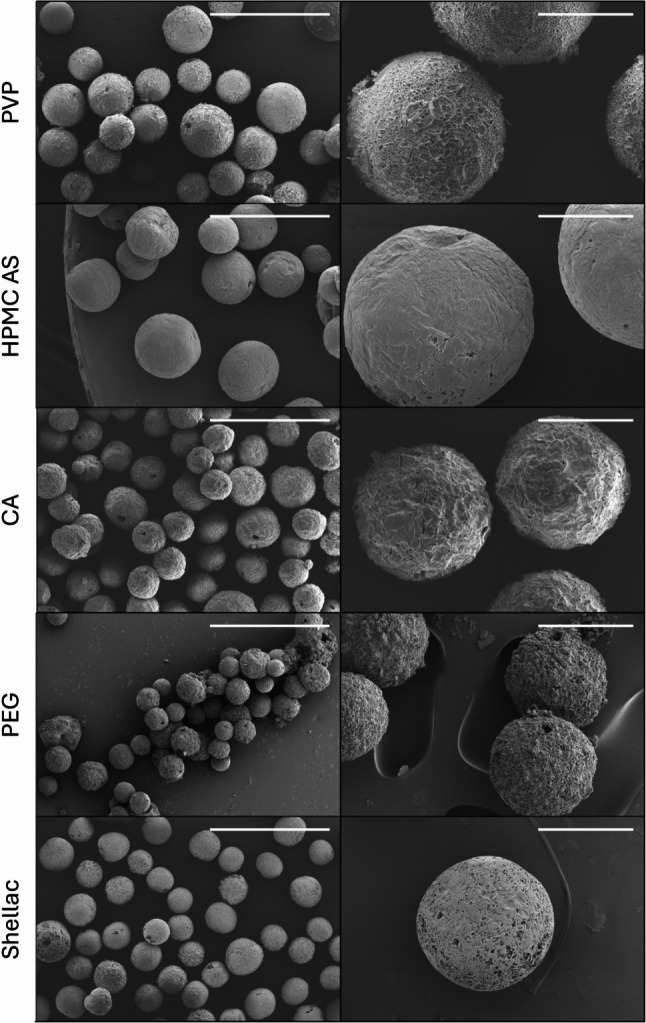
SEM images of spray freeze dried spheres in a resin: excipient ratio of 25:75, scale bars represent 1 mm (left panel) and 200 μm (right panel)

**Fig. 3:**
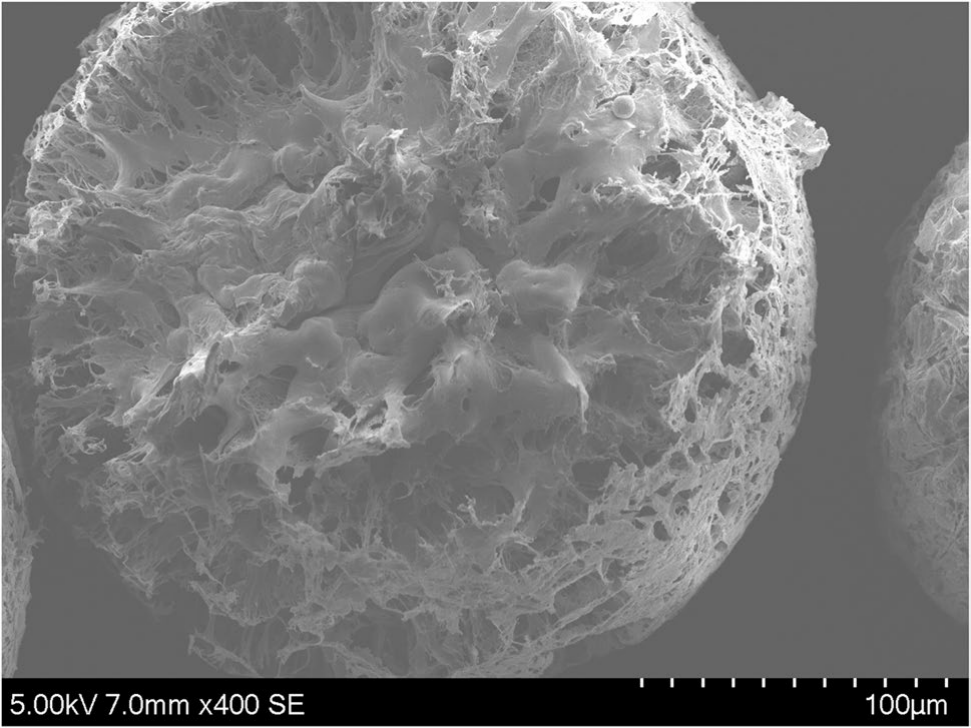
Fractured shellac cannabispheres reveals highly porous internal structure in combination with inner core collapse which directly translated in the reduction of mean particle diameters in the case of shellac cannabispheres

The pure tert-butanolic extract formed no cake but a semisolid resin on the bottom of the vial upon freeze-drying, as no structure-forming excipient was present. On the other hand, despite the semisolid nature of the resin, all the final spray-freeze-dried particles were solid, free-flowing, and spherical (Fig. 2). Only the PEG 6000 particles were not freely flowing and were slightly sticky already at room temperature. When stored at 40 °C, they softened and partially agglomerated and fused. Shellac-based cannabispheres had a porous appearance but also partially collapsed structure directly after preparation (Fig. 3), this remained unchanged over 12 months of storage at 4 °C; however, upon three months of storage at 25 °C, or after one month at 40 °C they collapsed completely into non-porous monolithic yellowish blocks. The PVP, HPMC-AS, and cetyl alcohol particles did not change in appearance even after 12 months at 40 °C.

**Fig. 4:**
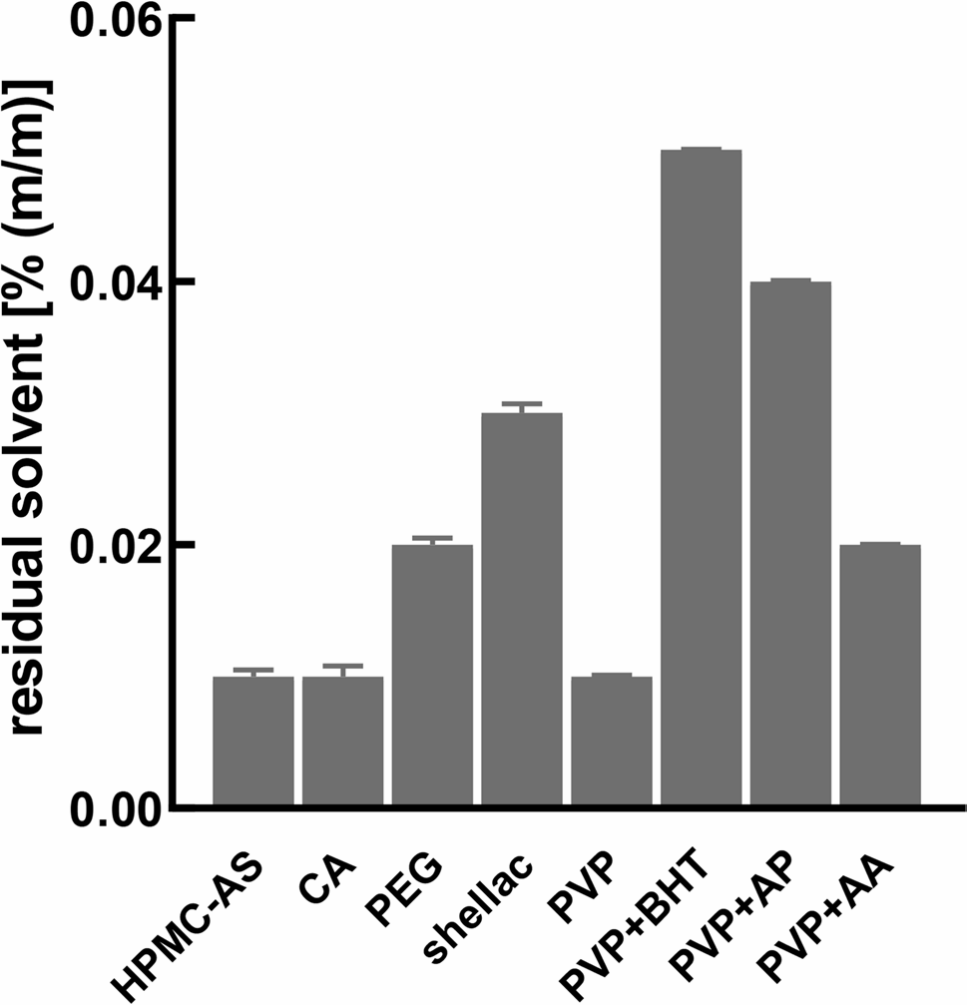
Residual solvent content in the various cannabispheres (data are given as means ± SD; *n* = 3)

The residual tert-butanol content was consistently below 0.1% (w/w) across all excipient formulations immediately after freeze drying (Fig. 4). The median particle size (D50) was 313 μm in case of PVP-spheres, 399 μm HPMC-AS, 261 μm cetyl alcohol, and 234 μm for shellac spheres.

### Long-term stability

**Fig. 5:**
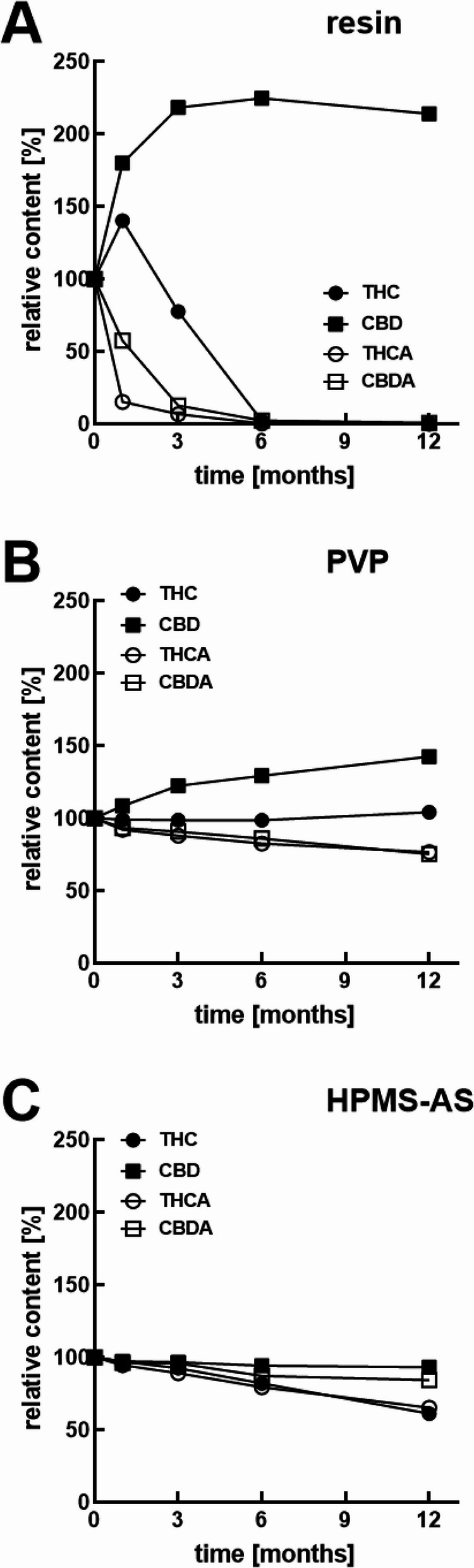
Long-term stability of the cannabinoids at 40 °C in pure resin (**A**), PVP- (**B**) or HPMC-AS-cannabispheres (**C**). Data are given as means ± SD (*n* = 3)

**Fig. 6:**
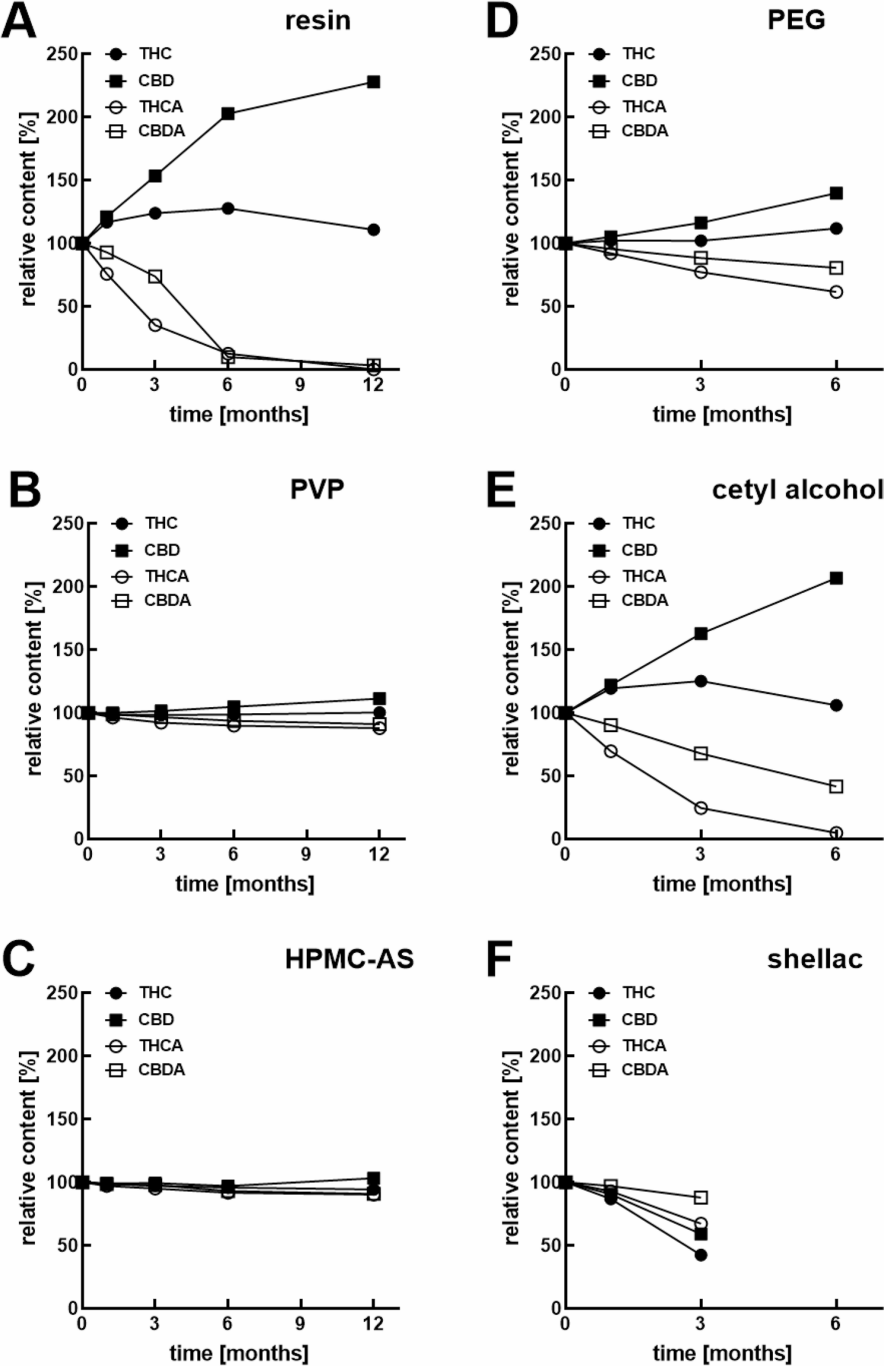
Long-term stability of the cannabinoids at 25 °C in pure resin (**A**), PVP- (**B**), or HPMC-AS- (**C**), PEG- (**D**), cetyl alcohol- (**E**), or shellac-cannabispheres (**F**). Data are given as means ± SD (*n* = 3)

When the dry crude resin was stored at 40 °C the content of THC and CBD increased after 1 month due to the formation of new entities of THC and CBD through the decarboxylation of THCA and CBDA (Fig. 5A). However, a strong decrease in THC was apparent thereafter, and after 6 months it was almost completely degraded. In the crude resin stored at 25 °C, the gradual THC degradation is still apparent despite masked by generation of new THC molecules from THCA (Fig. 6A), resulting in a net increase of 10.8% (*p* < 0.001). CBD content at 12 months increased significantly by 114.1% at 40 °C (Table S1) and 127.9% at 25 °C (Suppl. Table 2), indicating better stability of CBD than THC in crude resin.

In the PVP particle formulation, the content of THC remained relatively stable over the 12 months period at 25 °C (Fig. 6B) as well as at 40 °C (Fig. 5B), supported by a non-significant difference from the initial content at 25 °C (Table S2), and a significant increment of 6.4% at 40 °C (Table S1). The decrease of THCA (-12.0% at 25 °C and − 24.3% at 40 °C) suggest ongoing conversion to THC, which in turn, could have masked the degradation of the initially content of THC. This degradation was, however, less pronounced than in the case of the crude resin. The CBD content increased by 42.5% more when stored at 40 °C (*p* < 0.001), as more CBD molecules were generated from CBDA (which was reduced by 25.8%, *p* < 0.001), and from the potential oxidation of THC. A significant difference (*p* < 0.001) was found in the CBD content at 40 °C for 12 months between pure resin (114.1%) and PVP formulation (42.8%).

The HPMC-AS formulation also promoted a stabilising effect, although the stabilizing effect was slightly less expressed than with PVP. After 12 months, THC was decreased significantly by 5.8% at 25 °C (Table S2) and 39.2% at 40 °C (Table S1), while CBD was degraded significantly only by -3.0% at 25 °C and 7.1 at 40 °C. Accordingly, degradations of THCA of 8.9% at 25 °C and − 35.0% at 40 °C; and − 9.5% at 25 °C, and − 15.1% at 40 °C for CBDA were significant in all cases. Significant differences (*p* < 0.001) were found in the endpoint CBD content at 40 °C (93.0%), relative to both pure resin (214.1%) and PVP formulation (142.8%).

In contrast, the cannabinoid content after a 3-month storage at 25 °C (Table S3) changed significantly in case formulations of shellac, with all four cannabinoids degraded; and PEG 6000 and cetyl alcohol, both with increased THC and CDB content, and decreased THCA and CBDA content. Despite the similarity between the stability behaviour of cetyl alcohol to the pure dry extract resin alone (Fig. 6D-F), the differences were significant in all cases (*p* < 0.05).

**Fig. 7:**
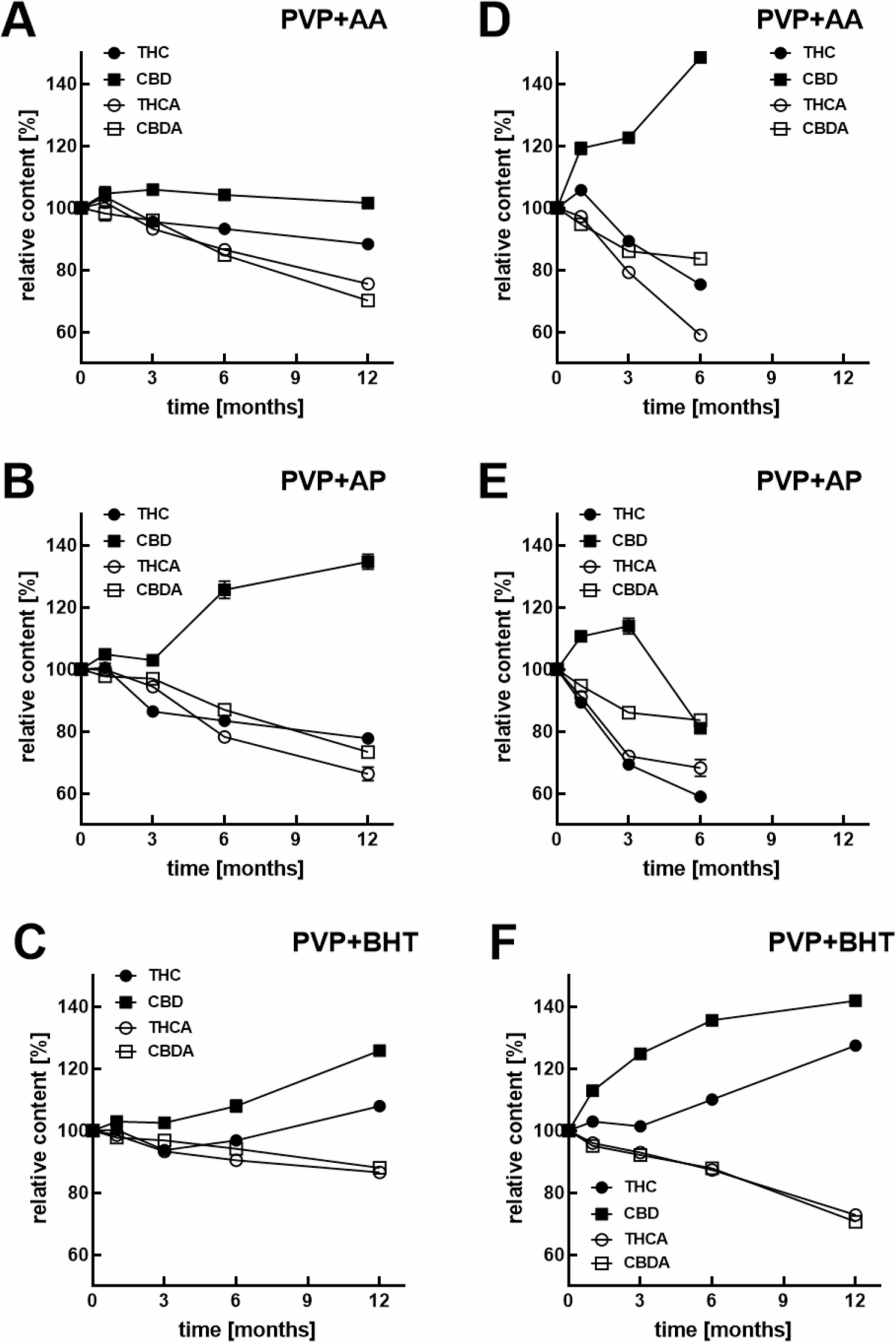
Long-term stability of the cannabinoids at 25 °C (**A**-**C**) or 40 °C (**D**-**F**) in PVP-cannabispheres and the respective effects of antioxidant additives, i.e. ascorbic acid (**A**, **D**), ascorbyl palmitate (**B**, **E**), or butyl hydroxytoluene (**C**, **F**). Data are given as means ± SD (*n* = 3)

The addition of neither of the antioxidants (BHT, ascorbyl palmitate or ascorbic acid in 5% of solid weight content) to the PVP formulation prevented the decarboxylation transformation of THCA to THC and CBDA to CBD in the SFD product. However, the BHT apparently prevented the subsequent degradation of the THC and CBD as the content of the both was higher than in pure PVP cannabispheres: after 12 months at 40 °C storage, THC increased to around 127% in spheres with BHT and only to around 104% THC in pure PVP while similarly it lead to an CBD content increase to around 172% of CBD with BHT vs. 142% CBD without BHT (Fig. 7D-F). At 25 °C, PVP BHT and PVP formulations exhibit a similar pattern in the degradation of THCA (with no difference, *p* = 0.793) and CBDA (more pronounced with BHT), meanwhile PVP AA and PVP AP showed a higher impact on this degradation. However, at these conditions only PVP, PVP AA and PVP BHT were able to maintain or increase the THC amount, compared to t_0,_ and only PVP and PVP AA showed a protective effect towards CBD content. Besides these protective effects, all remaining changes in the four cannabinoids compared to t_0_ were determined as significant (Table S1 + 2) and, apart from no differences in the final content of THCA (between PVP and PVP BHT, *p* = 0.072), and CBDA (between PVP BHT and PVP AA, *p* = 0.179), no further similarities were found between formulations with antioxidants incorporated.

**Fig. 8:**
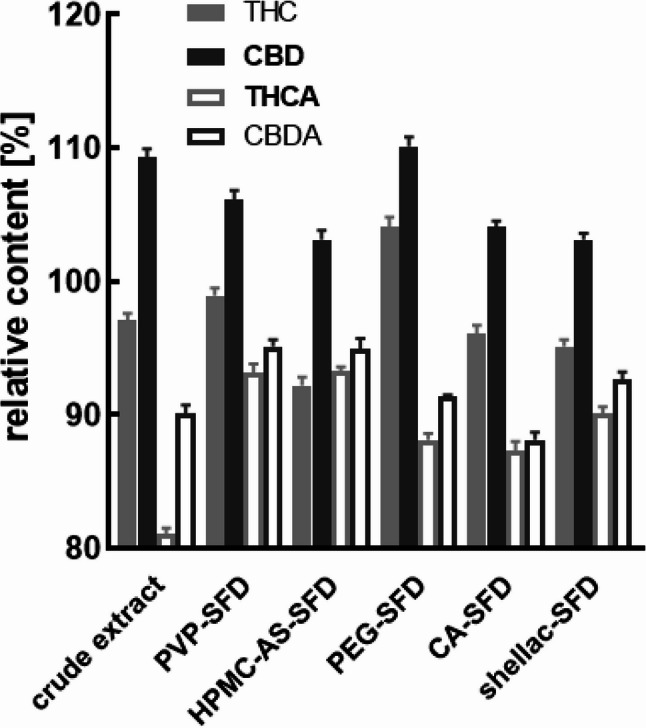
Long-term stability of the cannabinoids in pure resin versus various cannabispheres after 12 months at 4 °C. Data are given as means ± SD (*n* = 3)

**Fig. 9:**
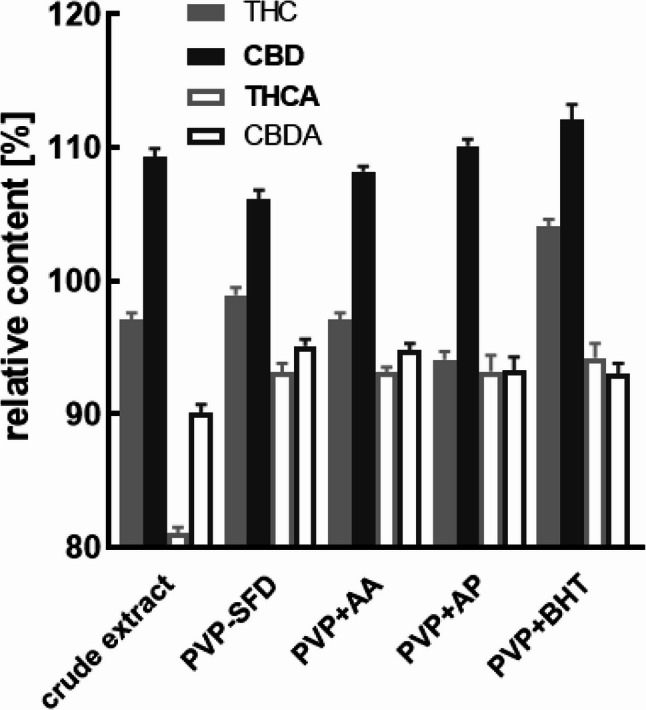
Long-term stability of the cannabinoids in pure resin versus PVP-cannabispheres and the respective effects of antioxidant additives, i.e. ascorbic acid (AA), ascorbyl palmitate (AP), or butyl hydroxytoluene (BHT) after 12 months at 4 °C. Data are given as means ± SD (*n* = 3)

When stored at 4 °C for 12 months, THC and CBD concentrations were in the range of 93 to 110%, but significantly different (Table S4) compared to t_0_, across all formulations and crude product. Distinctively, only PVP formulation showed no difference comparing to the content at t_0_ (*p* = 0.749). The observed CBD concentration always increased (*p* < 0.001) compared to t_0_. The acids THCA and CBDA in the crude extract showed the strongest degradation with 80 and 90% respectively. Despite all formulations exhibit different grades of degradation compared to t_0_ (*p* < 0.001), PVP and HPMC-AS formulations lead to less pronounced changes on the final amounts of the four cannabinoids. The addition of antioxidants did not affect the decarboxylation of the acids to the neutral form compared to the antioxidant free formulation, which is in accordance with the data presented after storage at 25 and 40 °C. In addition, the PVP BHT formulation was the only formulation that exhibited an increase in both THC and CBD concentration, which shows the strong protective potential of BHT against oxidative degradation of THC even after 1 year of storage (Table S4). This effect was observed across all temperatures, with increasing concentrations of both forms with increasing temperatures (Figs. 8 and 9).

### In vitro dissolution

**Fig. 10:**
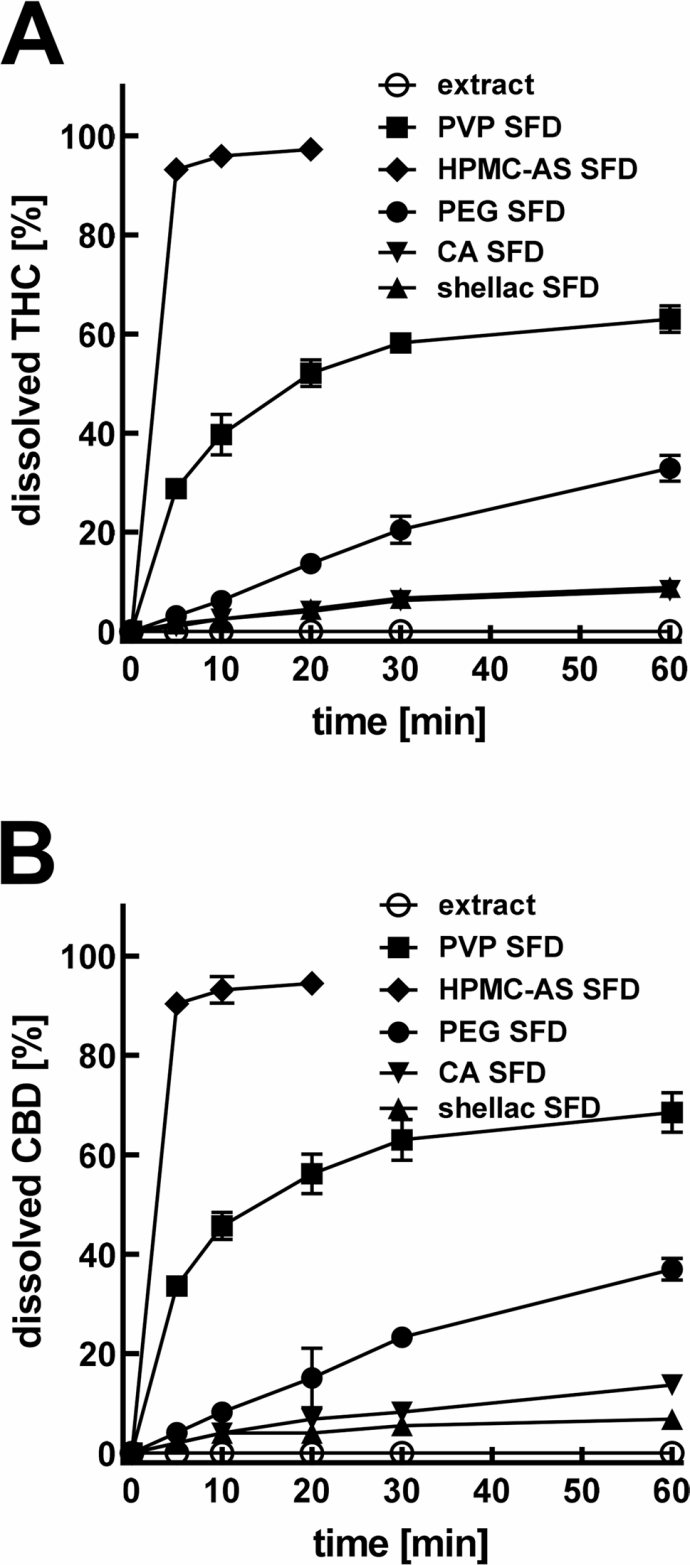
Dissolution test of cannabispheres in phosphate buffer at pH 7.0 containing 0.1% polysorbate 80. Cumulative release/dissolution is given in percent of initial THC (A) and CBD (B) content (means ± SD; *n* = 3)

The dissolution of THC and CBD from the pure crude resin reached 1.1 ± 0.6% of THC and 1.0 ± 0.4 of CBD, respectively, within 60 min (Fig. 10); 4.2 ± 0.9% THC and 5.8 ± 1.0% CBD after 12 h and a plateau at 5.6 ± 0.9% THC and 8.3 ± 1.1% CBD released was reached after 48 h.

Due to this poor dissolution performance of the pure resin, together with the low variability of groups, effect sizes (Cohen’s coefficient) were determined as large (d > 3.00) for every statistically different comparison.

The dissolution performance of the cannabispheres was strongly dependent on the excipient used, as confirmed by one-way ANOVA (*p* < 2 × 10^-16^ for both THC and CBD). Excipient type accounted for over 99% of the variance (effect sizes coefficients of η² = 0.9993 and ω² = 0.9989, for THC; and η² = 0.9989 and ω² = 0.9983, for CBD).

Cannabispheres formulated with hydrophobic excipients (cetyl alcohol and shellac) did not disintegrate noticeably during the dissolution testing and, accordingly, the amount of released THC and CBD was relatively low. After 60 min, cetyl alcohol cannabispheres released 8.2 ± 0.5% THC and 14.4 ± 0.4% CBD; while shellac cannabispheres released 7.4 ± 0.1% THC and 7.4 ± 0.2% CBD. No significant difference was found for THC dissolution between shellac and cetyl alcohol (*p* = 0.99; d = 1.82, 95% CI: 0.22–3.42). However, a significant large effect size of cetyl alcohol over shellac was found for CBD dissolution (*p* < 0.001).

Cannabispheres with hydrophilic polymers (PVP, PEG 6000 and HPMC-AS) were all able to strongly enhance the dissolution rate of THC and CBD. All three showed significantly enhanced dissolution (Tukey’s HSD: *p* < 0.0001 and large effect sizes: d > 8.00 for all of comparisons). The HPMC-AS cannabispheres dissolved rapidly, releasing 97.8 ± 1.1% of THC and 94.8 ± 1.5% of CBD content within 20 min. PVP cannabispheres reach a dissolution of 63.5% THC and 68.2% CBD at 60 min. PEG 6000cannabispheres gradually agglomerated during the dissolution and merged into larger aggregates and, despite the hydrophilic nature of PEG 6000, showed intermediate performance, releasing 32.7 ± 2.5% THC and 37.4 ± 2.3% CBD. Pairwise comparisons among the hydrophilic excipients confirmed significant differences (Tukey’s HSD: *p* < 0.0001 and large effect sizes: d > 16.98).

## Discussion

The cannabis extract is a semisolid and sticky resin upon removal of the extraction solvent, which makes it difficult to handle. The formulation with further excipients via spray-freeze-drying enabled its conversion into solid, non-sticky, free-flowing particles. Similarly, pure THC has also a semisolid consistency as the resin, and a precedent study on freeze-drying of pure THC together with inulin as a structure-forming excipient has been described (Van Drooge et al. 2005). Tert-butanol was successfully employed here as a solvent for non-aqueous spray freeze drying. Although is listed by the ICH classification as a class 2 solvent, the very low residual solvent content in the final particles being less than 0.1% (w/w) in all cannabispheres confirms to fit into these regulations. The similarity of extraction profile of tert-butanol compared to ethanol as it has been demonstrated here, enables the use of tert-butanol already in the extraction step, hence reducing the number of process steps and solvents involved in the whole procedure. Moreover, we were able to show that this extends the application of the SFD process to a whole cannabis extract.

The choice of the excipients for cannabispheres formation is generally limited by their sufficient solubility in tert-butanol and as found in a recent study also impacted by the resulting viscosity of the spray solution (Lucas et al. 2022; Kožák et al. 2025).

The structural stability, especially outer morphology and porosity, of the spheres with incorporated cannabis resin was apparently determined by the glass transition temperature (T_g_) of the structure-forming excipient. Shellac has a low T_g_ of around 40–50 °C (Farag et al. 2009) and the plasticizing effect of the incorporated resin was likely responsible for the gradual collapse of the porous structure during storage. The PVP with a T_g_ ≈ 156 °C and HPMC-AS with a T_g_ ≈ 120 °C correspondingly formed mechanically robust, non-sticky particles that remained structurally stable over the whole period of investigation, even at 40 °C. The higher T_g_ of PVP and HPMC-AS can also likely explain the improved chemical stability of the incorporated cannabinoids.

However, different factors may be involved in the case of PEG 6000 and cetyl alcohol particles. While both possess low melting point - PEG 6000 around 60 °C and cetyl alcohol around 50 °C - the PEG 6000 cannabispheres were sticky already at room temperature whereas the cetyl alcohol particles remained stable even at 40 °C, did neither collapse, nor got sticky, nor melted. Therefore, not just the melting point per se seems to play a decisive role. The hydrophobic cannabis resin is not miscible with the hydrophilic PEG and phase separation and migration of the resin to the surface of the freeze-dried particles could have occurred, resulting in the sticky appearance, poor flowability, and poor chemical stability. On the other hand, the hydrophobic cetyl alcohol was able to better accommodate the alike hydrophobic resin (Hommoss et al. 2017), without phase separation; hence, leading to structurally more stable and non-sticky lyophilisates. Nevertheless, the storage stability of the incorporated cannabinoids was poor with both excipients.

A generally poor chemical stability of cannabinoids is the major challenge in essentially all formulations being the principal reason for their limited shelf-life. According to the official summary of product characteristics of the commercially available Sativex^®^ oromucosal spray, the product must be stored in a fridge and upon opening it may not be used longer than 42 days. Similarly, ethanolic solutions of CBD were found stable at 4 °C, but the content decreased to approx. 80% after 12 months at 25 °C and to approx. 45% after 9 months at 40 °C (Fraguas-Sánchez et al. 2020).

Cannabis oil extracts, another widely used formulation type, also show limited cannabinoid stability (Carcieri et al. 2018). In olive oil extract, stored for 14 days at ambient temperature, the THC content decreased to 72% and content of other cannabinoids to around 80% (Pacifici et al. 2017). The CBD in sunflower oil degraded to approx. 25% of the initial content after one year when stored in closed vials at 40 °C and to nearly 0% in open vials; and around 60% after one year in closed vial at 25 °C (Kosović et al. 2021). THC-loaded solid lipid nanoparticle suspension was developed earlier as a nasal spray (Hommoss et al. 2017); and a good chemical stabilisation of THC was achieved (79% of the THC remained after 6 months of storage at 40 °C). However, such formulations still bear the general disadvantages of aqueous suspensions such as the physical, hydrolytic, and microbial instabilities. Besides, the authors found that approx. 10% of the initial THC content degraded during the preparation process (hot-high-pressure homogenisation) (Hommoss et al. 2017).

Even inferior stability was found in aqueous solutions, e.g. in decoction the entire THC content degrades within 14 days (Pacifici et al. 2017). Also CBD showed poor stability in aqueous solution (+ 0.5% polysorbate 80) at 25 °C degraded to 50% after 19 days, in simulated physiological conditions at pH 7.4 and 37 °C degraded to 50% after 7 days (Fraguas-Sánchez et al. 2020).

As many other compounds, cannabinoids exhibit higher stability in solid state than in solutions. Accordingly, pure crystalline CBD was observed to be more stable (after 1 year at 40 °C decrease to 90%) than the pure amorphous THC (completely degrades after 50 days at 20 °C) (Kosović et al. 2021; Van Drooge et al. 2005).

The presented results confirm that storage stability of the cannabinoids is strongly depended on the formulation. In the crude resin, THC degraded almost completely after 6 months at 40 °C, while CBD showed better stability, approximately doubling over the same period due to conversion from CBDA. Spray-freeze-dried particles stabilized with PVP or HPMC-AS significantly reduced cannabinoid degradation, with PVP providing the strongest protection: THC remained relatively stable and CBD increased steadily. Other excipients (PEG 6000, shellac, cetyl alcohol) showed poor stabilization, comparable to crude resin. It could be hypothesised that the improved stabilization with PVP and HPMC-AS may not only be attributed to their high Tg but also involves specific polymer–cannabinoid interactions, such as hydrogen bonding with hydroxyl groups of cannabinoids, as demonstrated recently for CBD in spectroscopic studies (Buczek et al. 2025). Whether this mechanism can sufficiently explain the observed increased stability of the CBD would need additional confirmation (e.g. via DSC, XRPD, FT-IR or solid-state NMR) which is however beyond the scope of the current study.

The addition of antioxidants did not prevent the decarboxylation of THCA and CBDA into THC and CBD. However, BHT markedly improved the stability of the neutral cannabinoids. After 12 months at 40 °C, PVP-BHT cannabispheres showed higher contents of THC and CBD compared to antioxidant-free PVP, demonstrating effective protection against oxidative degradation. At 4 °C, all formulations remained largely stable, though CBD consistently increased while the acids declined. The stabilizing effect was unique to BHT and not observed with ascorbic acid or ascorbyl palmitate.

While our analysis focused on the major cannabinoids THC, CBD, THCA, and CBDA, it should be noted that minor cannabinoids and terpenes may also contribute to the overall therapeutic profile. Their evaluation was beyond the scope of this study and could be considered in future work to more comprehensively assess full-spectrum extract formulations.

The poor solubility and slow dissolution of THC and CBD from the pure resin, attributed to the hydrophobic nature of the resin, was strongly improved by formulating them in the form of the SFD particles, which dissolve/disintegrate rapidly even at low fluid volume as found e.g. at the nasal mucosa (Serim et al. 2021). HPMC-AS spheres achieved the fastest dissolution for THC as well as for CBD; making it suitable for applications where rapid dissolution is desirable, e.g. for applications via the nasal or sublingual administration route. Although the cannabispheres exhibited a geometric size of 230–400 μm, their highly porous, low-density, and friable structure, composed of mucoadhesive HPMC/PVP, is expected to alter their functional behavior compared to solid particles of similar size. Such properties can potentially facilitate rapid wetting and dissolution, while also supporting mucosal adhesion relevant for oromucosal application, where sprays were found superior to oral capsules in reduction of neuropathic pain (Meng et al. 2017).

Especially in the context of processing polymers, the SFD process allows for more facile control of both, size and shape of the powders produced, when e.g. compared with spray drying (Saluja et al. 2010; Ali et al. 2014; You et al. 2024). In SFD, both parameters mainly depend on the size of the primary droplet before freezing and their stability upon lyophilisation. As the solvent removal occurs when the particles are in solid state, harder to control complex transport phenomena as e.g. accumulation of polymer in the shell leading to early crust formation, collapse of particles, occurrence of irregular shapes, and consequently poor flow properties of the powders produced and control of the particle size distribution, are inherently avoided. Not involving heat, the SFD process can be especially suitable for processing the heat-sensitive cannabinoids (Van Drooge et al. 2005).

Moreover, the reduced effective aerodynamic diameter of these aggregates may enhance their suitability for nasal delivery. In line with this, recent studies with SFD-produced PVP and HPMC particles prepared with TBA and loaded with melatonin (250–350 μm) achieved > 95% nasal deposition across all formulations and doses in a nasal deposition analyser (Rautenberg et al. 2024).

In SFD particles typically two solubility-enhancing strategies can be combined, i.e. the incorporation in a matrix of a hydrophilic polymer together with the intrinsically large surface area of the lyophilisates that is accessible for dissolution. This usually leads to an increased dissolution rate, often in combination with a supersaturation (Pacifici et al. 2017). The role of the large surface area and the porosity of the spheres is further underlined by the significantly accelerated release from cetyl alcohol cannabispheres compared to the pure crude semisolid resin, despite the rather hydrophobic nature of the cetyl alcohol.

## Conclusion

Spray freeze dried particles of *Cannabis flos* full extract, formulated in this study with either hydrophilic or lipophilic carrier excipients, comprise interesting features of both, liquid formulations, i.e. increased dissolution rate, and solid-state formulations, i.e. improved storage stability.

The non-aqueous spray-freeze-drying process does not involve any heat stress and has proven to be a suitable process for the formulation of a heat-sensitive cannabis full extract. Remarkably, the entrapment of cannabinoids in the hydrophilic polymers PVP or HPMC-AS considerably improved both the long-term stability of THC and CBD, and their dissolution rate and extent.

## Supplementary Information

Supplementary Material 1.

## Data Availability

The authors declare that the data supporting the findings of this study are available within the paper. Should any raw data files be needed in another format they are available from the corresponding author upon reasonable request.
