# Supplementary material for: Combined extraction and spray-freeze-drying of Cannabis sativa Flos provides stable lyophilizate particles of cannabinoids
Source: J Cannabis Res. 2026 Jan 6;8:16. doi: 10.1186/s42238-025-00381-w (PMC12837605; doi:10.1186/s42238-025-00381-w)
Supplement: Supplementary file 1 — Supplementary Material 1. [file 42238_2025_381_MOESM1_ESM.docx]

**Supplementary materials**

**Table S1. Changes in cannabinoid content relative to t_0_ after 12 months at 40 °C**

|  | Difference (%) | | | | | |
| --- | --- | --- | --- | --- | --- | --- |
| Cannabinoid | Pure resin | PVP | HPMC-AS | PVP AA | PVP AP | PVP BHT |
| THC | -99.9 | +6.4 | -39.2 | -24.6 | -41.2 | +27.4 |
| CBD | +114.1 | +42.8 | -7.1 | +48.8 | -19.8 | +72.0 |
| THCA | -+100.0 | -24.3 | -35.0 | -40.7 | -33.1 | -27.2 |
| CBDA | -99.3 | -25.8 | -15.1 | -30.1 | -16.8 | -29.3 |

p < 0.001 in all cases

**Table S2. Changes in cannabinoid content relative to t_0_ after 12 months at 25 °C**

|  | Difference (%) | | | | | |
| --- | --- | --- | --- | --- | --- | --- |
| Cannabinoid | Pure resin | PVP | HPMC-AS | PVP AA | PVP AP | PVP BHT |
| THC | +10.4 | 0^‡^ | -5.8 | 11.4 | -22.5 | +8.3 |
| CBD | +127.9 | +11.6 | +3.0 | 0^‡^ | -33.5 | -26.4 |
| THCA | -100.0 | -12.0 | -8.9 | -24.2 | -34.8 | -13.0 |
| CBDA | -96.7 | -8.8 | -9.5 | -29.3 | -26.9 | -11.6 |

‡: p > 0.1; non-marked values: p < 0.001

**Table S3. Changes in cannabinoid content relative to t_0_ after 3 months at 25 °C**

|  | Difference (%) | | | | | |
| --- | --- | --- | --- | --- | --- | --- |
| Cannabinoid | Pure resin | PVP | HPMC-AS | PEG 6k | CA | Shellac |
| THC | +23.9 | +1.4** | -2.9 | +2.1** | +25.1** | -57.7 |
| CBD | +53.5 | +1.7** | 0‡ | +16.3 | +62.7 | -41.0 |
| THCA | -64.8 | -7.9 | -5.1 | -22.8 | -75.5 | -32.7 |
| CBDA | -26.1 | -3.2 | -2.3 | -11.4 | -32.3 | -12.2 |

CA: cetyl alcohol; ‡: p > 0.1; **: p < 0.05; non-marked values: p < 0.001

**Table S4. Changes in cannabinoid content relative to t_0_ after 12 months at 4 °C**

|  | Difference (%) | | | | | | | | |
| --- | --- | --- | --- | --- | --- | --- | --- | --- | --- |
| Cannabinoid | Pure resin | PVP | HPMC-AS | PEG 6k | CA | Shellac | PVP AA | PVP AP | PVP BHT |
| THC | -2.7 | 0‡ | -8.1 | +4.4 | -3.9 | -4.8 | -2.6 | -6.2 | +4.3 |
| CBD | +9.5 | +6.6 | 0‡ | +10.2 | +4.1 | +3.2 | +8.5 | +9.9 | +12.5 |
| THCA | -18.8 | -6.4 | -6.8 | -11.7 | -12.7 | -9.8 | -6.6 | -7.4 | -5.4 |
| CBDA | -9.7 | -4.6 | -5.5 | -8.5 | -11.9 | -7.3 | -4.8 | -7.2 | -6.7 |

CA: cetyl alcohol; ‡: p > 0.1; non-marked values: p < 0.001
